# Psychological Needs and Problematic Social Media Use in Adolescents: A Gender-Moderated Mediation via Sensation Seeking and Cognitive Flexibility

**DOI:** 10.3390/healthcare14010008

**Published:** 2025-12-19

**Authors:** Kübra Dombak, İbrahim Erdoğan Yayla, Samet Makas, Eyüp Çelik, Ümit Sahranç, Mehmet Kaya

**Affiliations:** 1 Faculty of Education, Guidance and Psychological Counseling, Sakarya University, 54050 Sakarya, Türkiye; kubra.dombak1@ogr.sakarya.edu.tr (K.D.); eyupcelik@sakarya.edu.tr (E.Ç.); sahranc@sakarya.edu.tr (Ü.S.); mehmetkaya@sakarya.edu.tr (M.K.); 2 Faculty of Education, Guidance and Psychological Counseling, Bayburt University, 69000 Bayburt, Türkiye; ibrahimeyayla@bayburt.edu.tr

**Keywords:** problematic social media use, basic psychological needs, cognitive flexibility, sensation seeking, adolescents

## Abstract

**Background:** The purpose of this study is to examine the mediating roles of cognitive flexibility and sensation seeking in the relationship between basic psychological needs and problematic social media use. Furthermore, the moderating effect of gender on indirect effects has been examined. **Method:** The sample of the study consisted of 838 Turkish adolescents aged between 14 and 18 (46.2% female; Mean = 15.6, SD = 1.71). Participants completed the Bergen Social Media Addiction Scale, the Basic Psychological Needs Scale, the Cognitive Flexibility Scale, and the Brief Sensation Seeking Scale. Data were analyzed using structural equation modeling (SEM) with the AMOS 26.0 program. **Results:** A significant relationship was found between basic psychological needs and problematic social media use (r = 0.43, *p* < 0.001). Both cognitive flexibility and sensation seeking partially mediated this relationship in girls (*β* = −0.23, *p* < 0.001), while fully mediating it in boys (*β* = 0.03, *p* = 0.675). **Conclusions:** The findings suggest that problematic social media use in adolescents may be associated with cognitive flexibility and increased sensation-seeking tendencies stemming from unmet psychological needs, and that gender plays an important role in this relationship.

## 1. Introduction

Throughout history, humans have felt the need to communicate as social beings and have developed various tools to meet this need. Today, internet-based technologies have become a fundamental element of communication. Social media platforms and online games, in particular, offer individuals opportunities for entertainment, socialization, and self-expression [1]. However, excessive and uncontrolled use of these tools can lead to negative consequences in individuals’ psychological and social lives. Recent studies show that social media and online games have experienced rapid growth. According to current statistical reports, there are over five billion social media users worldwide [2,3]. In Turkey, this number is reported to be 58.50 million [2]. Although there is no current and official data on social media use among adolescents living in Turkey, the Information Technology Use in Children Survey, conducted by the Turkish Statistical Institute [4], with participants aged 6–15, determined that the social media usage rate is 66.1%. These rates reveal the interest of children and adolescents in social media applications. Social media has become an integral part of modern life, particularly among adolescents, and its prevalence has steadily increased [5,6]. In the new media age, social media use is an important form of social interaction for adolescents, shaping their social environments for learning and living, as well as offline interactions. However, prolonged and uncontrolled use of social media platforms negatively impacts adolescents’ physical health, psychological well-being, and behavioral patterns, paving the way for problematic social media use over time [7].

A significant portion of studies on internet-based addictions highlight the concept of problematic social media use. These studies draw attention to the addiction criteria discussed in the Diagnostic and Statistical Manual of Mental Disorders (DSM-5) published by the American Psychiatric Association. It is stated that addiction criteria such as anxiety, tolerance development, and loss of control apply not only to internet gaming disorder but also to problematic social media use. In the DSM-5, “Internet Gaming Disorder” is addressed under a separate heading and classified in the third section, which includes disorders requiring further research [8]. However, problematic social media use is not included as a diagnostic category in the DSM-5. Limiting internet-based addictions to gaming disorder alone may lead to the neglect of other behavioral addictions related to internet use. Furthermore, due to the lack of a standard definition in this field, studies examining the relationship between social media and mental health use terms such as ‘social media addiction’ [9,10,11], ‘problematic social media use’ [12,13], and ‘social media use disorder’ [14,15]. Studies suggest that excessive social media use should be considered a behavioral addiction [16,17].

According to generally accepted definitions in the literature, problematic social media use is characterized by an individual’s intense interest in social media platforms, a desire to use them that they find difficult to control, and the negative impact of the time spent on these platforms on their daily life responsibilities and social relationships [18]. This suggests that social media use can progress from a simple habit to a behavioral addiction. Research suggests that this type of use can have negative effects on the psychological and physical well-being of adolescents [19,20]. In addition to psychological consequences such as decreased self-esteem and increased symptoms of anxiety and depression, it has also been reported that risks such as online aggression, self-harming behaviors, suicidal thoughts, and poor sleep quality can increase [20,21]. Not only cognitive processes but also emotional regulation and how psychological needs are met play a decisive role in the development of problematic social media use. A study by Quaglieri et al. [22] demonstrated a strong relationship between emotion regulation and problematic social media use. The researchers suggested that emotional dysregulation, through fear of missing out (FoMO), exacerbates problematic social media use, transforming it into a form of emotional regulation strategy for individuals. This finding suggests that social media serves as a “digital relaxation space,” particularly for individuals experiencing emotional instability. The concepts of sensation seeking and cognitive flexibility are also critical in understanding problematic social media use in adolescents. Varchetta et al. [23] conducted a study with a Spanish sample, finding that males use social media more problematically due to impulsivity and high stimulation, while females were found to be fueled by emotional sensitivity and low self-esteem. Another study conducted by the same research group demonstrated that the effects of emotional regulation difficulties and FoMO on problematic social media use can be explained by an indirect mediation model [24]. These findings suggest that the psychological processes underlying problematic social media use interact with variables such as gender, emotional sensitivity, and stimulation seeking. Furthermore, boredom (trait boredom) has also been identified as a significant trigger for problematic social media use.

A systematic review by Tagliaferri et al. [25] found that individuals turn to social media due to internal emptiness and low levels of offline stimulation; this behavior creates a cyclical reinforcement mechanism, perpetuating addiction. For adolescents, social media becomes a tool that provides both emotional stimulation and escape. A broader psychological framework underlying these processes can be explained by the “emotional reinforcement mechanism” developed by Wang and Wang [26]. According to this model, problematic social media use is maintained not only through extrinsic rewards (e.g., likes, comments) but also through the internal reinforcement of positive emotions and the suppression of negative ones. In this sense, social media serves as a digital platform for emotional regulation, particularly for adolescents. Consequently, problematic social media use should be considered not simply as a technological habit but also as a consequence of emotional regulation difficulties, high sensation seeking, low cognitive flexibility, and how psychological needs are met in adolescents. The interaction of emotional and cognitive processes provides a fundamental framework for understanding how both risk and protective factors come into play in the development of this addiction.

## 2. Literature Review

### 2.1. Basic Psychological Needs and Problematic Social Media Use

Social networks enable individuals to post updates at any time of the day via phones, computers, and tablets, while also keeping them informed about innovations in social media applications [27]. Bayer and colleagues [28] explain the functioning of social media through four basic components: profile, communication network, feed, and messaging. Within this framework, users first create a profile and share content, then develop a communication network by following various people, interact by viewing other users’ posts through the feed, and maintain communication through messaging. The multidimensional opportunities offered by social networks have garnered global attention, making social media one of the indispensable elements of communication today [29]. Social media is not only a tool for information sharing and interaction, but also affects individuals’ emotions and basic psychological needs. Yee [30] defined the emotions arising from social media use based on three factors: platform immersion, success, and social factors. These factors are seen to be related to individuals’ basic psychological needs, such as feeling independent and adequate.

How adolescents meet their psychological needs, interests, and personal orientations has a decisive impact on their developmental processes [31]. The social content offered by social media also plays a significant role in helping individuals form relationships. The individual’s need to be accepted by their peer group, the desire to maintain their presence in the virtual world, and the need to escape stressful situations are among the primary motivations for increased social media use [32]. It has been observed that individuals who feel excluded from their social circles, experience feelings of inadequacy, or feel fear develop friendships through social media and distance themselves from face-to-face social interaction in the process [33]. Furthermore, it has been noted that individuals’ experiences of failure and environmental conditions are also important factors that drive them to social media [34]. These findings suggest that how psychological needs are satisfied plays a crucial role in the development of problematic social media use.

The fundamental psychological needs addressed within the Self-Determination Theory refer to the requirements of autonomy, competence, and relatedness that must be met for individuals to experience health, happiness, and well-being [35]. Autonomy refers to an individual’s ability to make their own choices freely; competence refers to an individual’s belief that they have the skills and resources to cope with the situations they encounter; and relatedness refers to the need to form meaningful bonds with others [36]. The literature emphasizes that these needs are universal in nature and play a decisive role in individuals’ personal and social development and behavior [37,38]. The fulfillment of basic psychological needs is defined as “need satisfaction,” while their non-fulfillment is defined as “need frustration.” It is stated that when needs are not sufficiently met, mental health is negatively affected and psychopathological risks increase [39]. Indeed, research shows that individuals whose needs are met tend to exhibit higher levels of well-being, life satisfaction, motivation, social adjustment, and academic achievement.

In contrast, those whose needs are not met are more prone to low motivation, academic failure, depression, anxiety, loneliness, and substance use [40,41,42]. As Deci and Ryan [35] also point out, individuals who are unable to satisfy their basic psychological needs adequately tend to resort to various means to fulfill these needs. Today, social media use is at the forefront of these means.

Social media can fulfill the need for autonomy, approval, and feedback mechanisms by providing individuals with the opportunity to express themselves and reflect on their decisions, as well as the need for competence, online interactions, and a sense of belonging. However, fulfilling these needs through social media may provide short-term pleasure but increases the risk of addiction in the long term. Research shows that while social media use is a functional tool for meeting basic psychological needs, excessive and uncontrolled use can lead to unmet needs, low life satisfaction, and psychological problems [39,41]. When examining studies on the relationship between problematic social media use and basic psychological needs, Bozkurt [43] and Nigar [44] found a negative correlation between the fulfillment of university students’ needs for autonomy, competence, and relatedness and problematic social media use. In his study, Sözen [45] showed that the satisfaction of basic psychological needs does not play a formative role in the relationship between personality traits and problematic social media use, but that the level of addiction increases with decreasing need satisfaction. Fard and colleagues [46] found that unmet basic psychological needs increase Instagram addiction and decrease psychological well-being, while Saeed and colleagues [47] found that individuals with unmet needs participate more in social media. Additionally, Gugliandolo et al. [48] found that unmet basic psychological needs predict problematic social media use, while Ponnusamy et al. [49] demonstrated that the need to connect with others significantly increases the likelihood of Instagram addiction. Therefore, based on the findings, problematic social media use can be considered a result of the unhealthy fulfillment of basic psychological needs.

### 2.2. The Mediating Role of Cognitive Flexibility

One of the most fundamental factors guiding human behavior is the level of fulfillment of psychological needs. In particular, the needs for autonomy, competence, and relatedness are key components that determine an individual’s psychological well-being and adjustment [42]. The fulfillment of these needs increases psychological well-being, life satisfaction, and coping skills in individuals, while their non-fulfillment leads to stress, helplessness, and maladaptive coping behaviors. The level of fulfillment of basic psychological needs affects not only emotional processes but also cognitive processes [50]. Cognitive flexibility is particularly noteworthy in this context. Cognitive flexibility is defined as an individual’s capacity to adapt to changing conditions, consider different perspectives, and reorganize their thoughts or behaviors when necessary [51].

Individuals with high cognitive flexibility tend to be able to take responsibility, make sense of their experiences, be entrepreneurial, and build more secure relationships with their social environment [52]. This is because cognitive flexibility requires the effective use of perception, memory, planning, and behavioral processes, thereby increasing individuals’ capacity to manage events related to their environment [51]. Indeed, it is positively associated with positive outcomes such as happiness [53], creativity [54], and academic achievement [55], while it is negatively associated with online gambling addiction [56], smartphone addiction [57], problematic social media use [58], anxiety, and depression [59]. Cognitive flexibility is also a fundamental component of executive functions, requiring individuals to switch between strategies to adapt to changes in task demands [60]. This process is closely related to cognitive processes such as concentration, decision-making, goal-directed focus, organizational skills, and inhibitory control [61]. These functions are heavily engaged in individuals’ social media use today [62]. However, it has been found that impairments in executive functions play a critical role in internet-related problems, including problematic social media use [63].

Individuals with low cognitive flexibility struggle to implement their goal-oriented plans, are unable to regulate their behavior flexibly, and have difficulty diverting their attention away from negative stimuli [64]. Indeed, various studies have shown that individuals with internet addiction have impairments in executive functions [65]. Furthermore, studies conducted on children and adolescents show that low cognitive flexibility is associated with higher levels of externalization problems (e.g., physical aggression and verbal bullying) [66]. In short, low cognitive flexibility can lead individuals to seek short-term and superficial solutions. In this context, when examining social media use, it is observed that individuals who struggle to meet their needs and have limited cognitive flexibility tend to turn to social media platforms more frequently to address their problems or fill their emotional void. Social media has become an attractive escape route because it offers individuals instant rewards, quick feedback, and opportunities to form relationships, albeit superficial ones [67]. However, this process may increase the risk of problematic social media use in individuals in the long term. In this regard, cognitive flexibility can be considered as a mediator in the relationship between basic psychological needs and problematic social media use.

### 2.3. The Mediating Role of Sensation Seeking

Adolescents often seek an exciting life, and social media can provide them with new experiences, diverse lifestyles, and opportunities for adventure. This can help fulfill adolescents’ need for self-expression and visibility [68]. In other words, adolescents may engage in sensation-seeking behavior through social media and seek to satisfy their craving for excitement. Posting on social media, accessing different content, and following constantly changing agendas can provide teens with a variety and dynamism they cannot find in real life [69]. Therefore, it can be considered that teens may attempt to meet their psychological needs and satisfy their sensation-seeking through social media. Research on problematic social media use has shown that addiction is related to both psychological needs and the level of sensation seeking [43,44,70]. In their study, Dursun and Eraslan-Çapan [71] showed that insufficient fulfillment of basic psychological needs increases addictive behaviors. Similarly, Taşbaşı and colleagues [70] found that individuals with high levels of sensation-seeking also have higher tendencies toward addiction. Individuals with higher sensation-seeking behavior are more eager to experience new things on social media, communicate with different people, and seek out attention-grabbing content [72]. These individuals may place less importance on the potential risks of social media use (such as time loss, loneliness, or declining academic performance) and focus on short-term gratification. Thus, their pursuit of excitement and pleasure can overshadow their concerns and accelerate the process leading to addiction.

Failure to satisfy basic psychological needs such as competence, autonomy, and relatedness, especially during adolescence, fuels the search for excitement and increases social media use. Adolescents who cannot satisfy their need for competence may gain a sense of achievement by receiving likes or gaining followers on social media [73]. Individuals whose autonomy needs are not met may view social media as a space for free decision-making and self-expression. Adolescents who experience a lack of relatedness may also turn to social media more intensively to connect with peers and receive social support [74]. This situation suggests that failing to meet basic psychological needs can lead to problematic social media use, driven by the search for excitement. Indeed, studies show that social media use intensifies during adolescence, when the level of excitement seeking is at its highest [75,76]. During this period, social media becomes a tool that feeds excitement and adventure for adolescents through constantly updated content, rapid interaction, and attention-grabbing posts. In short, the failure to meet basic psychological needs increases sensation-seeking among adolescents; increased sensation-seeking, in turn, raises the risk of problematic social media use. In this context, it can be said that adolescents’ levels of sensation-seeking not only have a direct effect on problematic social media use but also play a mediating role in the relationship between basic psychological needs and addiction.

### 2.4. The Moderating Role of Gender

The level of satisfaction of basic psychological needs plays a decisive role in the relationship individuals establish with digital platforms. According to self-determination theory [42], failure to meet basic needs such as competence, relatedness, and autonomy may lead individuals to compensate for these deficiencies through external sources. In this context, social media can become an area that substitutes for psychological satisfaction, especially for adolescents. However, the strength and direction of this relationship can be shaped not only by individual differences but also by socio-cultural variables such as gender. Research shows that men exhibit a higher tendency toward problematic social media use, while women use these platforms more for social interaction and emotional sharing [77,78]. This differentiation suggests that not only the purposes of use but also the psychological processes leading to problematic social media use may vary by gender. At this point, the Dual System Model [79] offers a robust theoretical framework for understanding risk-taking tendencies unique to adolescence. According to this model, during adolescence, the socio-emotional system, based on reward sensitivity, matures early, while the cognitive control system (including impulse control, planning, etc.) develops more slowly. It is suggested that this developmental imbalance between the two systems is more pronounced in males, leading them to show greater sensitivity to environments that provide sudden and intense rewards [79].

On average, male adolescents tend to exhibit higher levels of sensation-seeking and risk-taking behavior [24,29,80], which may make them more susceptible to the fast-paced, innovative, and competitive content offered by social media. At the same time, some studies show that males exhibit lower levels of cognitive flexibility and emotional regulation skills compared to females [81,82,83]. This situation may cause men whose basic psychological needs are not sufficiently met to struggle to develop alternative coping strategies and to use social media more frequently as a dysfunctional escape mechanism. On the other hand, while the effects of sensation-seeking and cognitive flexibility are partially valid for adolescent girls, their social media use is based more on social and emotional foundations such as the need for relatedness, emotional expression, and seeking social approval [84,85]. This situation shows that different needs are at the forefront in the development of addictive behaviors. However, explaining gender differences solely in terms of cognitive or emotional processes may be insufficient. Cultural expectations, social roles, the purpose of time spent in the digital environment, and the way the need for social approval is shaped may also contribute to this difference [86,87].

In conclusion, gender emerges as an important moderating variable shaping the relationship between basic psychological needs and problematic social media use. The stronger mediating role of individual characteristics, such as cognitive flexibility and sensation seeking, can explain why this relationship is more pronounced and negative in males. In contrast, in females, this process is more balanced by social context and emotional needs.

### 2.5. Present Study

The increasing use of social media has introduced new risks, particularly during the developmental processes of adolescents. The intensification of the time adolescents spend on social media can trigger a series of problematic situations, including attention problems, a decline in academic performance, social withdrawal, and difficulties in regulating emotions. Therefore, problematic social media use is considered a developmental risk factor for adolescents [18]. The literature provides strong evidence that the level of satisfaction of basic psychological needs is decisive in this process; insufficient satisfaction of these needs increases adolescents’ social media use and paves the way for addiction [42,43,44]. Recent studies have shown that individual differences, such as cognitive flexibility and sensation seeking, play a significant role in social media usage patterns. It has been observed that adolescents with high cognitive flexibility can use social media more controlled, while those with low cognitive flexibility are more prone to addictive behavior patterns [45,46,52,66]. On the other hand, adolescence is also a period characterized by an intense search for excitement. It has been noted that adolescents, who are constantly seeking novelty and stimulation, are increasingly drawn to the rapid interaction and dynamic content offered by social media, and that this plays a role in increasing addiction [29].

**Figure 1:**
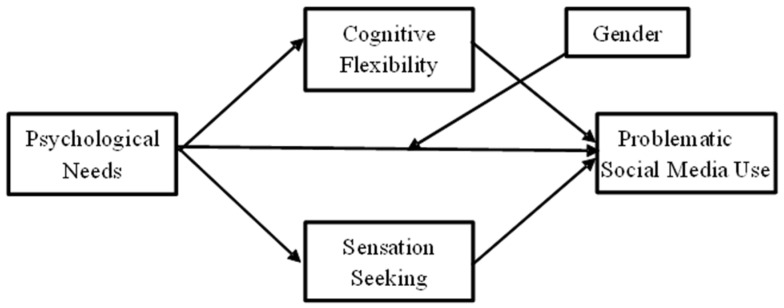
Hypothetical model.

Within this framework, the present study aims to examine the mediating role of cognitive flexibility and sensation seeking in the relationship between basic psychological needs and problematic social media use among adolescents. Furthermore, the study aims to comprehensively evaluate the risk and protective factors explaining problematic social media use by addressing these two individual characteristics within the same model. Additionally, the study examines the moderating role of gender. During adolescence, how girls and boys satisfy their psychological needs, their motivations for using social media, and their emotional responses may differ. These differences make it important to examine how gender creates a moderating effect on the mediating roles of cognitive flexibility and sensation seeking in the relationship between the satisfaction of basic psychological needs and problematic social media use. The variability of these relationships according to gender may indicate differences in the functioning of both risk and protective factors. In this respect, the study aims to contribute to the development of more sensitive and targeted intervention strategies for problematic social media use, taking into account not only individual differences but also gender-based differences. Social media plays a significant role in the lives of adolescents during this period. Therefore, studies on problematic social media use are also at a critical juncture. The creation of new roadmaps for research on problematic social media use, the strengthening of its theoretical foundation, and the examination of new elements necessitate the development of methods that will facilitate a better understanding of addiction [88]. In order to develop new methods and techniques related to problematic social media use, it is also crucial to increase the number of studies that address factors that may be related to addiction together. In this respect, this study is significant in that it addresses problematic social media use in adolescents not only in terms of need satisfaction but also in relation to cognitive and personality-based processes. Thus, it provides a theoretical basis for the development of early intervention and preventive strategies for problematic social media use, shedding light on psychoeducational programs that can be implemented, particularly in educational institutions and family environments. Furthermore, testing the mediating role of cognitive flexibility and sensation seeking, which have been addressed together in a limited number of studies in the literature, makes the study unique. The research hypotheses and the hypothetical model (Figure 1) are presented below.

**H1.**  *Basic psychological needs are associated with problematic social media use.*

**H2.**  *Cognitive flexibility mediates the relationship between basic psychological needs and problematic social media use.*

**H3.**  *Sensation seeking mediates the relationship between basic psychological needs and problematic social media use.*

**H4.**  *Cognitive flexibility and sensation seeking parallel mediate the effect of basic psychological needs and problematic social media use.*

**H5.**  *The indirect effects of psychological needs on problematic social media use through cognitive flexibility and sensation seeking differ according to gender.*

## 3. Method

### 3.1. Participants

The study was conducted on 838 Turkish adolescents, comprising 387 (46.2%) girls and 451 (53.8%) boys. Of the participants, 14.7% were 13 years old, 16.6% were 14 years old, 17.7% were 15 years old, 12.3% were 16 years old, 20.5% were 17 years old, and 18.3% were 18 years old (Mean = 15.6, SD = 1.71). The sample consists of students in grades 7 through 12 attending various state secondary schools and high schools across different regions of Turkey. During the data collection process, approximately 950 students were approached, and 921 students agreed to participate in the study, voluntarily completing the scales. Due to missing data, inconsistent responses, and incomplete parental consent forms, 83 forms were excluded from the analysis, resulting in a final sample of 838 participants.

Participants were selected using convenience sampling. This method was chosen due to its feasibility in light of the study’s access permissions to different schools, time constraints, and resource limitations. This approach facilitated reaching a broad student population and enhanced the study’s feasibility.

### 3.2. Measures

#### 3.2.1. Bergen Social Media Addiction Scale

The Bergen Social Media Addiction Scale (BSMAS) was used to assess the level of problematic social media use among adolescents [89]. The scale was adapted into Turkish by Demirci [90]. The scale is a 5-point Likert and is one-dimensional. It consists of a total of six items measuring six core symptoms of addiction (mental preoccupation, tolerance, withdrawal, mood change, conflict, and failed attempts). According to the CFA analysis results, the adaptation study showed a good fit (*χ*^2^/*df* = 1.33, SRMR = 0.04; RMSEA = 0.05, CFI = 0.99, TLI = 0.98, *p* = 0.214). In terms of reliability, the internal consistency coefficient (Cronbach’s α) of the scale was found to be 0.83 [90]. The Cronbach’s alpha value for this study sample is 0.85.

#### 3.2.2. The Basic Psychological Needs Scale

It was used to assess the extent to which adolescents’ basic psychological needs (relatedness, autonomy, and competence) are satisfied in their lives. The scale [Original: [35], Turkish: [91]] consists of 21 items, a 5-point Likert scale, and has three sub-dimensions (relatedness, competence, and autonomy). Higher scores on the scale indicate an increased desire for the relevant psychological need. In the external validity of the scale, significant correlations were found between the sub-dimensions of the Basic Psychological Needs Scale and Edwards Personal Preference Inventory (*r* = 0.36–0.58, *p* < 0.05). The internal consistency coefficients (Cronbach’s alpha) for the sub-dimensions were 0.73, 0.61, and 0.73 [91]. In the present study, Cronbach’s alpha values for the three subscales are 0.79, 0.75, and 0.81.

#### 3.2.3. Brief Sensation Seeking Scale

It is used to assess the level of sensation seeking in adolescents. The scale [Original: [92], Turkish: [93]] uses a 4-point Likert scale, consists of 4 items, and is one-dimensional. The validity of the scale was examined using EFA, and it was found that the one-factor structure explained 64.02% of the total variance. The internal consistency coefficient of the scale was 0.81, and the test–retest reliability was 0.84 [93]. The Cronbach’s alpha value for this study sample is 0.72.

#### 3.2.4. Cognitive Flexibility Scale

The Cognitive Flexibility Scale was used to assess cognitive flexibility in adolescents [Original: [94]; Turkish adaptation: [95]]. The scale consists of 12 items prepared on a 6-point Likert scale. Each core dimension contains three positive (flexible thinking) and three negative (rigid thinking) statements. High scores on the scale indicate that the individual has a high ability to adapt to situations and generate alternative solutions. In the Turkish adaptation study, confirmatory factor analysis revealed a good fit (*χ*^2^/*df* = 2.79, RMSEA = 0.06, CFI = 0.94, GFI = 0.91, SRMR = 0.07), and the scale’s internal consistency (Cronbach’s α = 0.87) was found to be high [96]. In this study, Cronbach’s α value is 0.91. Furthermore, the convergent validity of the scale has been supported in the literature; cognitive flexibility scores show positive correlations with problem-solving skills, self-regulation, and psychological well-being, and negative correlations with stress and anxiety levels [95,96]. These findings suggest that the scale is a valid measure of the target construct (Table 1).

### 3.3. Procedure

Prior to the data collection process, ethical approval was obtained from the Ethics Review Board of Bayburt University (Ethical Approval No: E-2025/054, Decision No: 54, Date: 20 February 2025). The research was conducted in accordance with the principles of the Declaration of Helsinki. Data were collected from various middle schools and high schools in different cities during May and June 2025. Participants consisted of adolescents aged 13 to 17 years. Participation was entirely voluntary, and written informed consent was obtained from parents for students under 18 years of age, along with consent (assent) from the students themselves. Participants were informed about the purpose of the study, and the principles of confidentiality and anonymity were strictly adhered to.

### 3.4. Data Analysis

This study was conducted to examine the mediating role of cognitive flexibility and sensation seeking, and the moderating role of gender in the relationship between basic psychological needs and problematic social media use among adolescents. In the first stage of the analysis process, the distribution characteristics and relational structures of the variables were evaluated using IBM SPSS Statistics 27 software. Descriptive statistics, including mean, standard deviation, skewness, and kurtosis values, along with Pearson correlation coefficients between variables, are presented in Table 2.

In the second stage of the statistical analysis process, a two-stage structural equation modeling (SEM) was applied to test the direct, indirect, and conditional relationships between variables. First, the measurement model was tested in the AMOS 26.0 program. Subsequently, based on the validated measurement model, a moderated mediation model was constructed, and the paths between variables were analyzed.

## 4. Results

### 4.1. Preliminary Analyses

Table 2 shows the correlations and descriptive statistics between problematic social media use, basic psychological needs sub-dimensions (autonomy, relatedness, and competence), cognitive flexibility, and sensation seeking.

Before proceeding to SEM analysis, correlation analysis between variables was examined (Table 2). Problematic social media use positively correlated with basic psychological needs sub-dimensions and sensation seeking. Problematic social media use was significantly negatively correlated with cognitive flexibility. Basic psychological needs sub-dimensions were also significantly negatively correlated with cognitive flexibility and positively correlated with sensation seeking. Furthermore, cognitive flexibility was significantly negatively correlated with sensation seeking.

When Table 2 is examined, it is seen that the skewness values of the variables are between −0.83 and 0.18, and the kurtosis values are between −1.17 and 0.24. The fact that these values are between −1.5 and +1.5 indicates that the distribution is within the limits that can be considered normal [97].

### 4.2. Structural Equation Model

In the study, the measurement model was tested before designing a structural equation model examining the mediating effects of cognitive flexibility and sensation seeking on the relationship between basic psychological needs and problematic social media use (*χ*^2^/*df* = 2.67, AGFI = 0.92, CFI = 0.95, GFI = 0.93, RMSEA = 0.04, TLI = 0.95, and SRMR = 0.04). Subsequently, the relationship between basic psychological needs and problematic social media use was examined.

**Figure 2:**
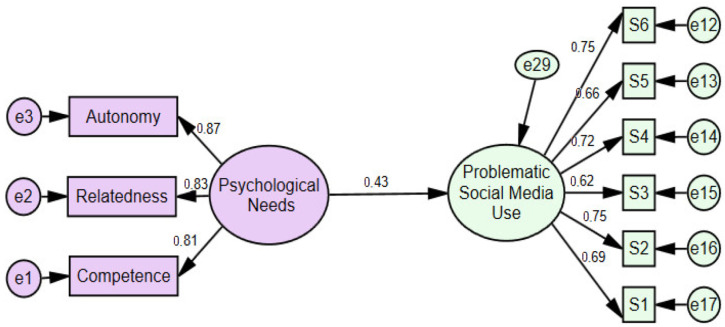
The total effect of psychological needs on problematic social media use.

When we examined the model in Figure 2, we found that the correlation between psychological needs and problematic social media use was (r = 0.43, *p* < 0.001). The fit indices for Figure 2 were found to be adequate with *χ*^2^/*df* = 1.91, SRMR = 0.05; RMSEA = 0.03, CFI = 0.99, TLI = 0.99, GFI = 0.99, AGFI = 0.98 [98,99].

### 4.3. Moderated Mediation Analysis

**Figure 3:**
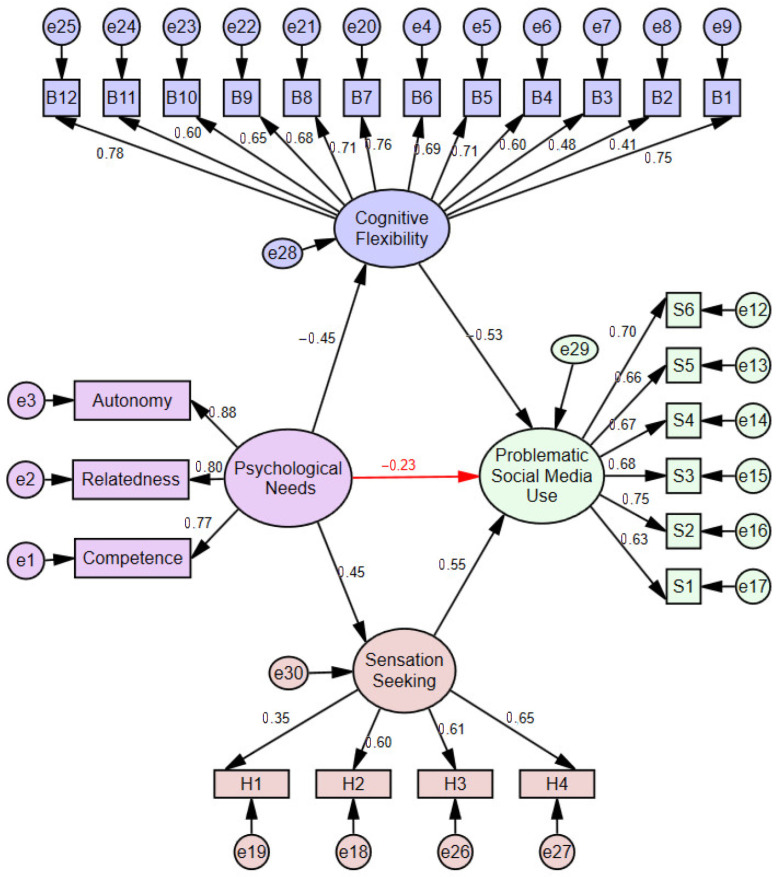
The outcome of the mediation model in girls.

**Figure 4:**
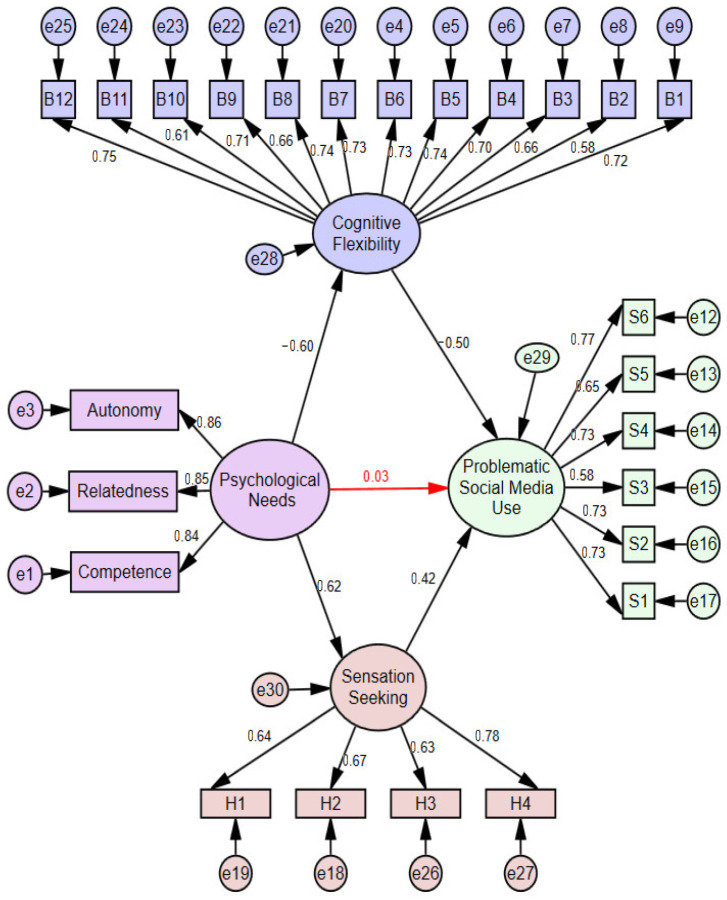
The outcome of the mediation model in boys.

The structural equation model was developed by including cognitive flexibility and sensation seeking as mediating variables in the relationship between psychological needs and problematic social media use. The model for girls is presented in Figure 3, while the model for boys is presented in Figure 4. The fit indices are shown in Table 3.

The regression coefficients of the structural paths that emerged in the model in Figure 3 with mediating variables were found to be statistically significant (*p* < 0.001). The structural model with sensation seeking and cognitive flexibility as mediators revealed that psychological needs significantly predicted cognitive flexibility (*β* = −0.45; *p* < 0.001). Similarly, the mediator variable, cognitive flexibility, significantly predicted the problematic social media use (*β* = −0.53; *p* < 0.001). It was revealed that psychological needs significantly predicted sensation seeking (*β* = 0.45; *p* < 0.001). It was revealed that sensation seeking predicted problematic social media use (*β* = 0.55; *p* < 0.001). When cognitive flexibility and sensation seeking were included in the relationship between psychological needs and problematic social media use, a direct effect of *β* = −0.23 (*p* < 0.001) was found in girls. The relationship between psychological needs and problematic social media use decreased from *β* = 0.43 to *β* = −0.23, and the level of the prediction decreased.

The regression coefficients of the structural paths that emerged in the model in Figure 4 with mediating variables were found to be statistically significant (*p* < 0.001). The structural model with sensation seeking and cognitive flexibility as mediators revealed that psychological needs significantly predicted cognitive flexibility (*β* = −0.60; *p* < 0.001). Similarly, the mediator variable, cognitive flexibility, significantly predicted the problematic social media use (*β* = −0.50; *p* < 0.001). It was revealed that psychological needs significantly predicted sensation seeking (*β* = 0.62; *p* < 0.001). It was revealed that sensation seeking predicted problematic social media use (*β* = 0.42; *p* < 0.001). When cognitive flexibility and sensation seeking were included in the relationship between psychological needs and problematic social media use, a direct effect of (*β* = 0.03; *p* = 0.675) was found in boys. The relationship between psychological needs and problematic social media use decreased from *β* = 0.43 to *β* = 0.03, and the direct effect became insignificant. The fit indices calculated to determine the statistical adequacy of the mediation model regulated by gender are shown in Table 3. Comparison of these values with the acceptance ranges specified in the literature revealed that the model has a satisfactory level of fit [98,100,101].

Table 4 summarizes the standardized coefficients and 95% confidence intervals for the direct and indirect effects of psychological needs on problematic social media use, based on 5000 bootstrap samples.

In summary, when cognitive flexibility and sensation seeking were included in the model, the predictive power of psychological needs on problematic social media use weakened in girls (partial mediation) but did not disappear entirely. In boys, it became statistically insignificant (full mediation). Additionally, the significance of the difference between groups according to the gender variable was evaluated using the “Critical Ratios for Differences” test. The analysis revealed a significant difference between the girl and boy groups in terms of path coefficients (*β*_girls_ = −0.23, *β*_boys_ = 0.03, C.R. = 2.579 > 1.96, *p* < 0.001). This finding indicates that the relationship between psychological needs and problematic social media use differs significantly according to gender.

**Figure 5:**
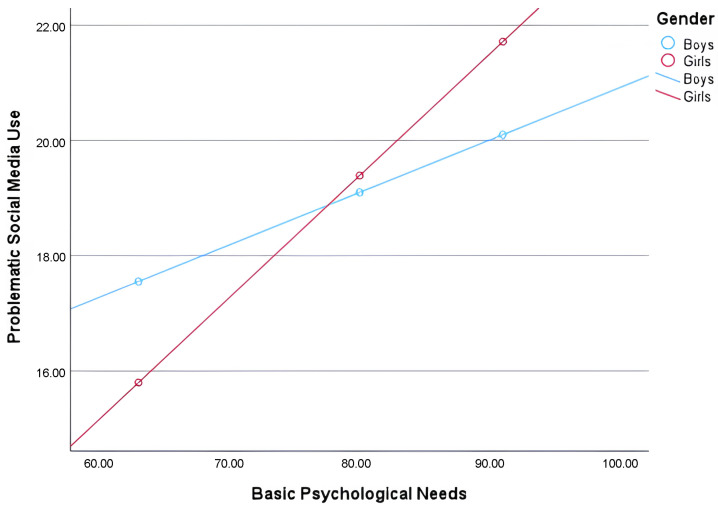
Interaction between psychological needs and problematic social media use as a function of gender.

Figure 5 shows that gender moderates the relationship between psychological needs and problematic social media use. As the level of psychological needs increases, problematic social media use rises more rapidly among girls than among boys.

## 5. Discussion

This study examined the relationship between adolescents’ basic psychological needs and problematic social media use and investigated the mediating role of cognitive flexibility and sensation seeking in this relationship. The findings revealed that cognitive flexibility and sensation seeking play a mediating role in the relationship between adolescents’ basic psychological needs and problematic social media use. The study results were evaluated in light of the relevant literature.

### 5.1. Basic Psychological Needs Are Associated with Problematic Social Media Use

The current study, consistent with recent research, suggests that the inadequate fulfillment of basic psychological needs may be associated with problematic social media use. The findings revealed positive and significant correlations between basic psychological needs, particularly competence, autonomy, and relatedness, and problematic social media use. These results are consistent with theoretical approaches suggesting that social media platforms provide individuals with opportunities to meet these needs [46,49]. For example, a study on Instagram addiction found that increased fulfillment of autonomy, competence, and relatedness needs was positively associated with tendencies toward Instagram use. In contrast, this was negatively associated with psychological well-being. These results suggest that social media use may function not only as an escape strategy but also as a platform through which individuals attempt to meet their basic psychological needs [46]. Similarly, a study conducted with university students showed that basic psychological needs are positively correlated with problematic social media use, and that this addiction may also be linked to academic procrastination [102]. These findings suggest that problematic social media use may not only be a consequence of need deprivation but also a reflection of individuals’ efforts to meet these needs in digital environments. Another study conducted in the context of romantic relationships reported that high levels of satisfaction of psychological needs such as love/belonging, power, and freedom are positively correlated with problematic social media use and phubbing [103]. Furthermore, research with adolescents has shown that poor social relationships and unmet basic psychological needs are positively correlated with problematic social media use [104]. Therefore, the current findings suggest that problematic social media use may be influenced not only by individuals’ unmet needs in offline environments but also by their tendency to satisfy these needs in virtual environments.

### 5.2. Cognitive Flexibility Mediates the Relationship Between Basic Psychological Needs and Problematic Social Media Use

The current study demonstrates that cognitive flexibility plays a significant role in the relationship between the level of fulfillment of basic psychological needs and problematic social media use. Findings suggest that individuals are more likely to exhibit behaviors associated with problematic social media use when their psychological needs are not adequately met; however, this relationship may vary depending on the individual’s level of cognitive flexibility. These results suggest that cognitive flexibility can be considered an important moderator or mediator variable in the processes associated with problematic social media use. Research demonstrates that low cognitive flexibility is positively associated with addictive tendencies. Studies conducted with university students have shown that low cognitive flexibility is significantly associated with problematic social media use and that cognitive flexibility partially explains this relationship [105,106]. Fard et al. [46] found a positive relationship between the unmet basic psychological needs and problematic social media use, indicating that individuals’ coping strategies are weakened in this situation. Lin et al. [104] found a negative relationship between the unmet basic psychological needs and decreased cognitive flexibility in adolescents, demonstrating that this relationship is also linked to problematic social media use. These results suggest that cognitive flexibility may be a potential protective variable in processes related to addictive behaviors.

Studies with university students have found that cognitive flexibility plays a mediating role in the relationship between problematic social media use and psychological well-being. Consequently, higher cognitive flexibility can attenuate the negative relationship between problematic social media use and psychological well-being [107]. Similarly, cognitive flexibility has also been shown to partially mediate the relationship between problematic social media use and academic procrastination [108]. Lower cognitive flexibility in students with higher problematic social media use appears to be positively associated with academic procrastination. The role of cognitive flexibility in various behavioral and emotional outcomes associated with problematic social media use has also been examined in different contexts. For example, Tanhan et al. [109] demonstrated that cognitive flexibility has both a moderating and partial mediating effect on the relationship between problematic social media use and phubbing behavior. Wang et al. [110] reported that cognitive flexibility functions as a variable that can moderate the negative relationship between problematic social media use and depressive symptoms. Taking these findings together, it can be argued that the relationship between the level of basic psychological need fulfillment and problematic social media use varies depending on the level of cognitive flexibility. Because individuals with high cognitive flexibility can also meet their needs offline, they exhibit weaker associations with excessive social media use.

### 5.3. Sensation Seeking Mediates the Relationship Between Basic Psychological Needs and Problematic Social Media Use

The findings of the current study indicate that there are significant relationships between the level of basic psychological need fulfillment and problematic social media use, and that sensation seeking plays a significant role in this relationship. When needs such as self-efficacy, autonomy, and relatedness are not sufficiently met, individuals seek stimulating, innovative, and exciting experiences, often finding these on social media platforms. These results suggest that social media use, rather than being solely a communication-based activity, serves the function of satisfying individuals’ psychological needs and seeking stimulation. Studies in the literature also support this relationship. For example, Meng et al. [72] found that sensation seeking is positively associated with problematic social media use among university students, with “fear of missing out” (FoMO) playing a partial mediating role in this relationship. According to the study, individuals high in sensation seeking feel a constant need for exposure to new and stimulating content, and this appears to be positively associated with their level of social media use. Similarly, Chaboki et al. [111] reported that sensation seeking is positively associated with internet addiction, and that this relationship is indirectly mediated through individuals’ level of self-enhancement. The same study found that this relationship was stronger in individuals with low self-efficacy.

A similar pattern was observed in a study conducted with older individuals. Cui et al. [112] reported that sensation seeking and “fear of missing out” played a sequential mediating role in the relationship between loneliness and excessive social media use. This finding suggests that problematic social media use is not limited to the younger population and that sensation seeking may be associated with similar psychological processes across different age groups. The findings suggest that low levels of basic psychological need fulfillment are positively associated with problematic social media use; this relationship may vary depending on individuals’ level of sensation seeking. Individuals with high sensation seeking appear more likely to meet their psychological needs in digital environments, such as social media, and this may be related to their social media usage patterns. Therefore, strategies that focus on balancing and regulating individuals’ sensation-seeking tendencies can make a significant contribution to interventions aimed at preventing problematic social media use.

### 5.4. Cognitive Flexibility and Sensation Seeking Mediates the Relationship Between Basic Psychological Needs and Problematic Social Media Use

The findings of this study indicate that there are significant relationships between the level of fulfillment of basic psychological needs (autonomy, competence, and relatedness) and problematic social media use, and that cognitive flexibility and sensation seeking act as parallel mediating variables in this relationship. The findings suggest that when psychological needs are not adequately met, individuals may exhibit lower levels of cognitive flexibility, and their pursuit of stimulating and innovative experiences may intensify. This suggests that individuals use social media environments not only as a means of social interaction but also as a stimulating space through which they attempt to satisfy their psychological needs indirectly. The literature also supports this relationship pattern. Numerous studies show that cognitive flexibility is negatively associated with problematic social media use and is an important variable in maintaining psychological well-being [105,107,109]. Çekiç and Öz [107] found that low cognitive flexibility was positively associated with problematic social media use; Damirchi et al. [113] reported that high sensation seeking, combined with low cognitive flexibility, is positively correlated with the tendency to engage in digital games. These findings suggest that as cognitive flexibility decreases, individuals’ tendency to seek out externally stimulating content may increase. Another study by Meng et al. [72] revealed that sensation seeking is positively correlated with problematic social media use, and that the constant novelty, instant feedback, and high levels of arousal offered by social media are particularly appealing to individuals with high levels of these tendencies. This suggests that problematic social media use is associated not only with behavioral but also with emotional reinforcement processes.

Recent neuroscientific studies indicate that dopamine-mediated reward circuits may underlie these psychological processes. Likes, comments, and shares received on social media trigger the release of dopamine in the brain’s reward center, creating a powerful reinforcement mechanism for continued use [114]. These neurochemical cycles demonstrate that problematic social media use is sustained through positive emotional reinforcement in the early stages and the avoidance of negative emotions in later stages [26]. Furthermore, in recent years, cognitive fatigue, distraction, and mental exhaustion associated with excessive social media use have begun to be referred to as “brain rot.” This phenomenon suggests that excessive consumption of low-quality content is linked to impaired executive function, emotional desensitization, and cognitive overload [115]. These findings reveal that the cognitive processes associated with problematic social media use have not only psychological but also neurophysiological underpinnings. Social rewards, such as likes and comments obtained in social media environments, create emotional reinforcement in individuals, sustaining behavioral cycles that strengthen addiction. This process points to a complex mechanism in which positive and negative reinforcement cycles operate simultaneously [26]. In conclusion, the findings suggest that processes related to unmet basic psychological needs, cognitive flexibility, and sensation-seeking levels exhibit theoretically consistent relationships with problematic social media use. This model offers a comprehensive theoretical framework for understanding how individuals’ cognitive and emotional processes may be related to their social media use patterns when they are unable to meet their psychological needs directly.

### 5.5. Discussion of the Moderating Role of Gender

Research findings reveal that the mediating roles of cognitive flexibility and sensation seeking in the relationship between basic psychological need satisfaction and problematic social media use may differ by gender. In the structural model, cognitive flexibility and sensation seeking were found to play a full mediation role in this relationship for male adolescents. In contrast, they played a partial mediation role for female adolescents. These results suggest that gender-based differences, in addition to individual characteristics, may play an important role in the psychological processes associated with problematic social media use [24]. The complete mediation observed in male adolescents suggests that the processes associated with problematic social media use may be more closely linked to cognitive and personality-based mechanisms [78]. Boys’ higher developmental tendency toward sensation seeking and their tendency to behave more reactively to external stimuli at lower levels of cognitive flexibility may lead them to exhibit stronger relationships with problematic social media use [29,81]. This finding is consistent with the Dual Systems Model [79], which posits that adolescent boys are more sensitive to fast-paced and constantly changing digital stimuli. It appears that boys whose basic psychological needs are not adequately met may tend to seek external gratification through social media and be more attracted to the intense stimulation these platforms offer.

The partial mediation observed in female adolescents suggests that problematic social media use may be related not only to cognitive or personality-based processes but also to emotional and social motivations [84]. Girls are known to focus more on psychosocial needs such as affiliation, emotional sharing, and social approval in their social media use [85]. This suggests that variables such as belonging, approval, and social comparison, in addition to cognitive flexibility or excitement seeking, may also influence adolescent girls’ problematic social media use-related behaviors [23]. From this perspective, while inadequate fulfillment of basic psychological needs may be directly related to problematic social media use in girls, the effects of mediating variables are weaker. These gender-differenced mediating structures gain significance when evaluated from the perspectives of self-determination theory [35] and developmental psychology. Higher external arousal and risk-taking tendencies, as well as lower levels of emotional awareness, observed in boys, may potentiate the influence of cognitive and personality-based processes associated with problematic social media use [77,78].

In contrast, variables such as intrinsic motivation, need for social affiliation, and self-worth may diversify the pathways associated with problematic social media use in girls [84,85]. In conclusion, this study suggests that gender may influence the type and strength of cognitive and motivational processes mediating the relationship between basic psychological need satisfaction and problematic social media use. In boys, this process is more cognitive and arousal-based, while in girls, it is more social and emotional.

The finding of complete mediation in boys and partial mediation in girls in the current study does not fully align with the general trends revealed by previous research. Meta-analysis studies generally report that boys are more prone to internet game addiction and women to problematic social media use [116]. However, the more pronounced mediation effects related to problematic social media use in male adolescents in the current study can be explained by several possible factors. The fact that the sample group in this study consisted of young adolescents may have influenced the direction of the results. Social media platforms, especially in recent years, have incorporated gamified features (points, competition, reward systems), which can transform social media use into a game-like experience for male adolescents. In this context, social media can serve not only as a social sharing environment but also as a platform for digital stimulation and performance for boys. At the same time, previous research suggests that men’s cognitive flexibility declines more rapidly, and they are more likely to engage in sensation-seeking behavior in stressful situations [29,81]. Because the cognitive flexibility and sensation seeking examined in this study are directly related to personality-based factors, these processes may have shown stronger correlations in men. Therefore, the different pattern observed in this study can be considered not as a deviation from the general trend in the literature, but rather as a theoretical differentiation based on the subtype of addiction and the nature of the measured variables.

### 5.6. Contribution and Limitations

The study’s findings provide important theoretical contributions to the development of prevention and intervention programs that consider cognitive flexibility and sensation seeking in addressing problematic social media use among adolescents. The findings suggest that the level of basic psychological need fulfillment is significantly related to problematic social media use. Therefore, implementing structured programs in school counseling services to support students’ basic needs, such as a sense of belonging, autonomy, and competence, may have a protective effect. Furthermore, the study found that low cognitive flexibility was positively associated with problematic social media use. This finding suggests that psychoeducational programs that develop flexible thinking, alternative solution generation, and cognitive reframing skills may be beneficial for adolescents in enhancing their capacity to cope with stressful situations.

Furthermore, sensation seeking was found to be a significant predictor of problematic social media use. This result suggests that integrating sports, arts, or nature-based activities into school environments, which allow adolescents to meet their needs for novelty and stimulation in healthier ways, may be supportive. The study also found that gender, as a moderating variable, created differences. The findings suggest that gender-specific psychological processes may play a role in the development of problematic social media use. Therefore, programs focused on strengthening cognitive flexibility skills and structuring sensation seeking may be prioritized for male adolescents.

In contrast, for female adolescents, activities focused on social support, emotional awareness, and self-esteem may be more effective. Overall, this study presents a comprehensive theoretical framework for understanding the psychological mechanisms underlying problematic social media use by examining the relationships among psychological needs, cognitive flexibility, and sensation seeking. In this respect, the study demonstrates that problematic social media use is not merely a behavioral phenomenon but also a multidimensional process related to how psychological needs are met.

As with any scientific study, this research has certain limitations. Firstly, as the study has a cross-sectional design, it is not possible to establish causal relationships between variables. Future studies could use longitudinal and experimental designs to test causal links. Another limitation is that the data were collected based solely on adolescents’ self-reports. This carries the risk of respondent bias. Future research could obtain more comprehensive and valid data by including teacher, parent, or peer observations. Another limitation is that certain psychological variables known to be associated with problematic social media use (e.g., emotional regulation, resilience, anxiety, boredom, and fear of missing out) were not controlled for in the model. The literature has demonstrated that these variables are associated with social media use. Therefore, including such variables in future research will contribute to a more comprehensive understanding of the psychological mechanisms underlying problematic social media use. Finally, as this study was conducted with secondary school/high school students in a specific region of Turkey using convenience sampling and did not collect detailed sociodemographic data (e.g., SES, internet/device access, daily usage time) beyond age and gender, the generalizability of the findings to different socio-cultural contexts is limited. Therefore, validation studies using larger, probability-based samples from different regions and including detailed demographic profiling are recommended. The final limitation of the study is that being male may not be a necessary or practical solution for problematic social media use, as the mediating role of males was not statistically significant. Therefore, future research could focus more on factors other than gender in problematic social media use.

## 6. Conclusions

This study examined the mediating role of cognitive flexibility and sensation seeking, and the moderating role of gender in the relationship between basic psychological needs and problematic social media use in adolescents. The findings revealed that both cognitive flexibility and sensation seeking are important mediating mechanisms in understanding the effect of unmet basic psychological needs on problematic social media use. Specifically, the insufficient fulfillment of basic needs (autonomy, competence, and relatedness) is associated with lower cognitive flexibility and higher sensation-seeking in adolescents, which in turn increases the tendency towards problematic social media use. These results suggest that cognitive flexibility and sensation-seeking are among the psychological processes that contribute to the problematic nature of adolescents’ social media use. Furthermore, it has been demonstrated that gender plays a significant moderating role in the relationship between basic psychological needs and problematic social media use, mediated by cognitive flexibility and sensation-seeking. The complete mediation observed particularly in male adolescents indicates that social media usage is shaped more by cognitive and personality-based mechanisms.

In contrast, the partial mediation in female adolescents suggests that other psychosocial factors, such as emotional attachment, self-worth, and social approval, also play a decisive role in social media interactions. These findings emphasize the importance of focusing on increasing young people’s cognitive flexibility and addressing their sensation-seeking behavior through healthier means in interventions targeting problematic social media use. Future studies are recommended to examine different psychosocial variables such as self-esteem, social support, parental control, and peer influence within similar models. Furthermore, the use of longitudinal and experimental research designs would be beneficial in clarifying causal relationships more clearly. Finally, it should be remembered that preventive and developmental mental health services developed for problematic social media use must take into account not only individual differences but also motivational tendencies and cognitive skills.

## Figures and Tables

**Table 1:** Characteristics of the measurement tools used in the study.

Scale Name	Instrument (Original/Turkish Version)	Items	Response Scale	Reliability (α)	Validity Evidence	Justification for Use
Bergen Social Media Addiction Scale	[89,90]	6	5-point Likert	[90]; 0.85 (current)	*χ*^2^/*df* = 1.33, SRMR = 0.04; RMSEA = 0.05, CFI = 0.99, TLI = 0.98, *p* = 0.214	Brief, validated Turkish version of a widely used scale for adolescents
The Basic Psychological Needs Scale	[35,91]	21	5-point Likert	0.73, 0.61, 0.73 [91]; 0.79, 0.75, 0.81 (current)	Expert review (content validity); significant correlations with Edwards Personal Preference Inventory (criterion validity)	Widely used, valid, and reliable Turkish measure of autonomy, competence, and relatedness needs
Brief Sensation Seeking Scale	[92,93]	4	4-point Likert	0.81 [93]; 0.72 (current)	EFA: single factor structure, explained variance = 64.02%	Brief, psychometrically sound measure of sensation seeking validated for Turkish adolescents
Cognitive Flexibility Scale	[94,95]	12	6-point Likert	0.87 [96]; 0.91 (current)	*χ*^2^/*df* = 2.79, RMSEA = 0.06, CFI = 0.94, GFI = 0.91, SRMR = 0.07	Reliable, valid self-report tool for Turkish samples

**Table 2:** The results of the correlation analysis.

Variable	a	b	c	d	e	f
a: Problematic Social Media Use	1					
b: Autonomy	0.33 **	1				
c: Relatedness	0.33 **	0.73 **	1			
d: Competence	0.33 **	0.71 **	0.67 **	1		
e: Cognitive Flexibility	−0.59 **	−0.44 **	−0.41 **	−0.41 **	1	
f: Sensation Seeking	0.53 **	0.39 **	0.38 **	0.40 **	−0.41 **	1
Mean	19.96	25.75	30.58	21.38	40.29	12.27
SD	6.20	5.30	5.93	4.60	13.08	3.14
Skewness	−0.19	−0.66	−0.83	−0.57	0.18	−0.76
Kurtosis	−1.16	0.09	0.24	−0.12	−1.17	−0.43
Condition Index	1	6.32	11.89	20.42	23.62	24.62
VIF	-	2.28	2.42	2.30	1.37	1.32
Tolerance	-	0.373	0.413	0.435	0.728	0.757

** *p* < 0.01.

**Table 3:** Acceptance ranges for fit indices and fit indices obtained from the mediator model test.

İndices	Perfect Fit Limit	Acceptable Fit Limit	Scale Indices	Result
X^2^/DF	0–2.5	≤5	1.87	Perfect
RMSEA	≤0.05	≤0.08	0.03	Perfect
SRMR	≤0.05	≤0.08	0.06	Perfect
CFI	≥95	≥90	0.95	Perfect
GFI	≥90	≥85	0.91	Perfect
IFI	≥95	≥90	0.95	Perfect

**Table 4:** Standardized path coefficients and bootstrap results for the mediation model.

Path	Coefficient (*β*)	SE	95% CI	
Standardized Total Effect			Lower	Upper
Psychological Needs → Problematic Social Media Use (Boys)	0.58	0.04	0.498	0.663
Psychological Needs → Problematic Social Media Use (Girls)	0.25	0.06	0.124	0.373
Standardized Direct Effect				
Psychological Needs → Problematic Social Media Use (Boys)	0.03	0.07	−0.112	0.163
Psychological Needs → Problematic Social Media Use (Girls)	−0.23	0.06	−0.365	−0.114
Standardized Indirect Effect				
Psychological Needs → Sensation Seeking + Cognitive Flexibility → Problematic Social Media Use (Boys)	0.56	0.06	0.450	0.684
Psychological Needs → Sensation Seeking + Cognitive Flexibility → Problematic Social Media Use (Girls)	0.48	0.06	0.369	0.629

## Data Availability

Dataset available on request from the authors: The raw data supporting the conclusions of this article will be made available by the authors on request.
